# Explainable Machine Learning–Based Prediction of Progression-Free Survival in Prostate Cancer: Retrospective Cohort Study

**DOI:** 10.2196/91510

**Published:** 2026-10-05

**Authors:** Hein Minn Tun, Lin Naing, Owais Ahmed Malik, Muhammad Syafiq Abdullah, Thu Ta, Hanif Abdul Rahman

**Affiliations:** 1 Pengiran Anak Puteri Rashidah Sa'adatul Bolkiah Institute of Health Sciences Universiti Brunei Darussalam Bandar Seri Begawan, Brunei-Muara District Brunei Darussalam; 2 School of Digital Science Universiti Brunei Darussalam Bandar Seri Begawan Brunei Darussalam; 3 Faculty of Engineering and Computing Atlantic Technological University Donegal Ireland; 4 The Brunei Cancer Centre Jerudong Park Medical Centre Bandar Seri Begawan, Brunei-Muara District Brunei Darussalam; 5 Saw Swee Hock School of Public Health National University of Singapore Singapore Singapore

**Keywords:** prostate cancer, progression-free survival, explainable AI, machine learning, prognosis

## Abstract

**Background:**

Progression-free survival (PFS) is a critical end point in oncology, yet real-world applications of individualized, explainable machine learning (ML) predictions remain limited.

**Objective:**

This study aimed to develop and validate explainable ML models to predict PFS using retrospective data from a national prostate cancer cohort in Brunei Darussalam.

**Methods:**

We analyzed a retrospective cohort of 212 patients (478 longitudinal observations) treated at the Brunei Cancer Centre (January 2018 to December 2024). Clinical, laboratory, and treatment data were harmonized, with missing values imputed via extremely randomized trees. Longitudinal patterns were captured using a recurrent autoencoder to generate latent representations. We compared 4 modeling approaches: Cox proportional hazards, random survival forest (RSF), gradient boosting survival (GBS), and deep neural network survival models. Performance was evaluated using time-dependent area under the receiver operating characteristic curve (AUC), Harrell C-index, and integrated Brier score (IBS), with Shapley additive explanations (SHAP) used for interpretability.

**Results:**

RSF demonstrated improved discriminative performance and balanced calibration, achieving a C-index of 0.906 and AUCs of 0.941 and 0.917 at 4 and 5 years (IBS=0.0698). In contrast, the traditional CPH model performed poorly (C-index 0.531 and AUC 0.706 at 4 years and 0.833 at 5 years). Deep survival (AUCs of 0.941 at 4 years and 0.917 at 5 years, C-index 0.719, and IBS=0.0887) and GBS (AUCs of 0.765 at 4 years and 0.833 at 5 years, C-index 0.844, and IBS=0.0590) models showed moderate performance. SHAP analysis identified sodium, alanine aminotransferase, mean corpuscular hemoglobin, platelet count, and specific treatment categories as key drivers of increased progression risk.

**Conclusions:**

Tree-based ensemble approaches, particularly RSF integrated with SHAP, offer high accuracy for personalized risk stratification in prostate cancer. These findings highlight the potential of explainable ML to enhance clinical decision-making. However, external validation in a larger multi-institutional, multiomics dataset is required before routine clinical implementation.

## Introduction

Prostate cancer is the fourth most common cancer in men worldwide, and the incidence of prostate cancer is increasing in Africa, Asia, and Latin America and the Caribbean, raising public health concerns worldwide [1,2]. Global and regional analyses demonstrate that the highest incidence rates are reported in northern Europe, North America, and Australia, largely due to the widespread use of prostate-specific antigen (PSA)–based early detection systems, while mortality is higher in Africa, Asia, and Latin America, indicating the global disparity based on screening and diagnostic capacity [2-5]. In Brunei Darussalam specifically, the national and international cancer registry data demonstrate the increasing prostate cancer deaths in recent decades [6,7]. These epidemiological trends in prostate cancer highlight the need for locally adapted prognostic and management tools that can improve the prostate cancer care continuum in Brunei Darussalam.

Progression-free survival (PFS) and disease-free survival are widely used in prostate cancer clinical trials and observational studies to quantify the duration during which a patient remains free from radiographic or clinical disease progression after treatment [8-10]. PFS often serves as a pragmatic outcome in trials of systemic and targeted therapies, as it can be observed earlier than overall survival and may reflect the treatment effect on tumor control. According to PCWG (Prostate Cancer Working Group) criteria, PSA progression is defined as a confirmed ≥25% and ≥2 ng/mL increase from the PSA nadir (or from baseline after ≥12 weeks if no nadir is achieved), with confirmation to exclude PSA flare, while radiographic or clinical progression, if occurring earlier, takes precedence in determining PFS [11]. Additionally, the understanding of PFS extension for prostate cancer is viewed differently between patients and clinicians. Patients generally require a longer PFS benefit to consider treatment worthwhile and tend to prioritize PSA or radiological responses despite potential side effects, whereas clinicians place greater emphasis on balancing treatment-related toxicity with PFS and may recommend therapy even when the expected PFS gain is modest [12].

Over the past decade, machine learning (ML) methods, including gradient-boosted trees, random survival forests, and deep learning architectures, have been increasingly applied to survival prediction and progression modeling across many cancer types, including lung and prostate cancer [13-17]. Furthermore, multimodal data integration of clinical variables, laboratory tests, imaging and radiomics features, and molecular data to produce individualized risk estimates for progression or death often outperforms traditional statistical models in complex, high-dimensional datasets [17,18]. Despite these advances, most ML and deep learning models for prostate cancer survival prediction rely on cross-sectional or clinical trial datasets with short follow-up periods, limiting their applicability in real-world practice [19-21]. Additionally, explainability methods such as Shapley additive explanations (SHAP) play an important role in the clinical applicability of these models by providing attributions for individual predictions [18,22,23].

However, few studies have explored the sequential analysis of progression free survival with explainable methods to provide locally calibrated, interpretable predictions for progression-free outcomes. Additionally, no previous study in Brunei Darussalam has been conducted to develop interpretable ML survival models for prostate cancer progression. To fill these gaps, our study aimed to develop explainable ML models that predict PFS using retrospective clinical, laboratory, and treatment information from a national cohort in Brunei Darussalam. By developing locally tailored, AI-enabled PFS models, this study contributes to the growing body of evidence supporting the use of ML and deep learning in precision oncology, while also advancing AI-based clinical decision support systems to assist oncologists in prostate cancer management and enhance the continuum of care in Brunei Darussalam.

## Methods

### Study Design and Data Source

This retrospective cohort study included patients with prostate cancer treated at the Brunei Cancer Centre (TBCC), Jerudong Park Medical Centre (JPMC) between January 2018 and December 2024. The study was conducted in accordance with the TRIPOD-AI (Transparent Reporting of a multivariable prediction model for Individual Prognosis or Diagnosis adapted for Artificial Intelligence) reporting guidelines to enhance transparency, reproducibility, and clinical applicability (Multimedia Appendix 1). Eligible participants were male patients with a histologically confirmed diagnosis of prostate cancer who received at least 1 treatment modality at TBCC. Patients with secondary metastatic prostate cancer or multiple primary malignancies were excluded following clinical review by oncologists. In total, 212 eligible patients contributed 478 time-indexed observations, capturing longitudinal treatment pathways and disease progression, as illustrated in Figure 1. Demographic, clinical, and treatment data were extracted from multiple sources, including the Brunei Darussalam Healthcare Information and Management System, the JPMC laboratory database, and both electronic and manually documented clinical records.

**Figure 1:**
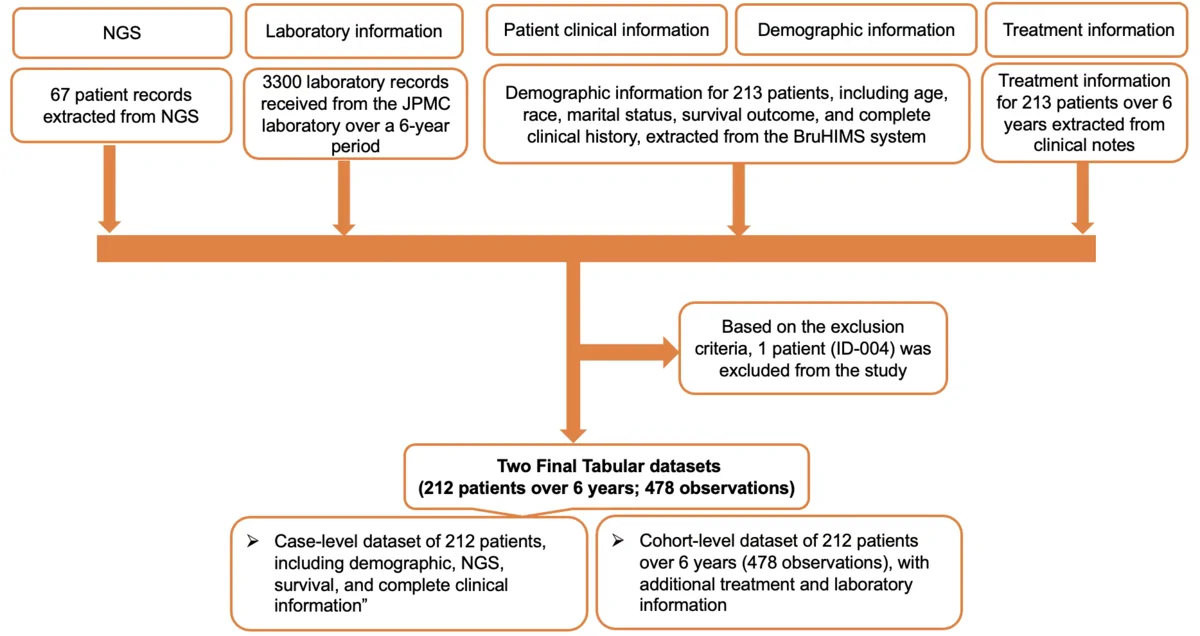
Overview of cohort data processing and source integration for the study. BruHIMS: Brunei Darussalam Healthcare Information and Management System; JPMC: Jerudong Park Medical Centre; NGS: next-generation sequencing.

### Data Structure and Outcome Definition

Data were structured across 2 hierarchical levels: the individual case level and the longitudinal cohort level. At the case level, collected variables encompassed demographic characteristics (including age, ethnicity, and marital status), medical history (eg, comorbidities and family history of malignancy), lifestyle factors (particularly smoking status), anthropometric measures (height, weight, and BMI), tumor-related features (Gleason score, tumor stage, and ECOG performance status), and survival outcomes. At the cohort level, comprehensive treatment information and 23 longitudinal laboratory measures were assembled, including baseline and 1-year posttreatment PSA values, full blood count parameters, liver and renal function indicators, erythrocyte sedimentation rate, calcium, and phosphate levels. Absolute and relative changes in PSA were derived, and tumor staging, Gleason grade groups, and National Comprehensive Cancer Network prognostic risk categories were harmonized to address inconsistencies in coding practices [24]. Except for PSA, all laboratory parameters were baseline measurements obtained before each treatment modification, enabling clinicians to assess laboratory results when determining subsequent therapeutic strategies. Data integrity was ensured through systematic cross-validation across multiple data sources and clinical verification by oncologists before analysis. The primary outcome was PFS.

PFS was calculated on a treatment-episode basis from treatment initiation to the earliest occurrence of PSA-defined progression, which was determined using PCWG-consistent criteria based on treatment-specific baseline PSA, posttreatment PSA nadir, and confirmed PSA rises (≥25% and ≥2.0 ng/mL, with timing and confirmation rules applied) or death from any cause, with patients censored at last follow-up in the absence of an event. A PFS event value of 0 indicates no biochemical progression or survival without progression at the last follow-up (censored), whereas a value of 1 indicates the occurrence of biochemical PSA progression or death from any cause. To conduct a survival analysis, PFS events, which denote the occurrence of progression or death, were used rather than PFS, which indicates the time a patient remains progression-free.

### Data Preprocessing and Survival Analysis

Statistical analyses were performed using RStudio (version 4.3.3; R Foundation for Statistical Computing), Python (version 3.10; Python Software Foundation) implemented via Google Colab, and Visual Studio Code (version 1.106.0; Microsoft). Two analytic datasets were created: a case-level dataset consisting of 212 patients and a longitudinal cohort-level dataset comprising 478 time-indexed observations. Data preprocessing procedures included data cleaning, harmonization of tumor staging and Gleason grade groups, elimination of duplicate records, and construction of patient-specific longitudinal sequences. At the case level, continuous variables were compared using the Mann-Whitney *U* test, while categorical variables were assessed using the Fisher exact test, with a significance threshold set at *P*<.05. For the cohort-level analysis, mixed-effects linear and logistic regression models were used to evaluate differences between survival groups. The differences between groups, including those stratified by comorbidity status, were assessed using the log-rank test.

### Data Splitting and Missing Data Handling

The overall ML model development pipeline is illustrated in Figure 2A. To minimize information leakage, data were split at the individual case level into training (80%) and testing (20%) sets using a random group-splitting strategy [25]. Missing values in both static and longitudinal variables were addressed using extremely randomized trees–based iterative imputation, a nonparametric approach derived from the random forest algorithm that accommodates mixed data types and captures nonlinear relationships [26]. Categorical variables were transformed using one-hot encoding, and continuous variables were standardized using *z* score normalization to ensure comparable feature scales. All preprocessing steps, including imputation, scaling, and encoder fitting, were performed solely on the training dataset and subsequently applied to the testing dataset.

**Figure 2:**
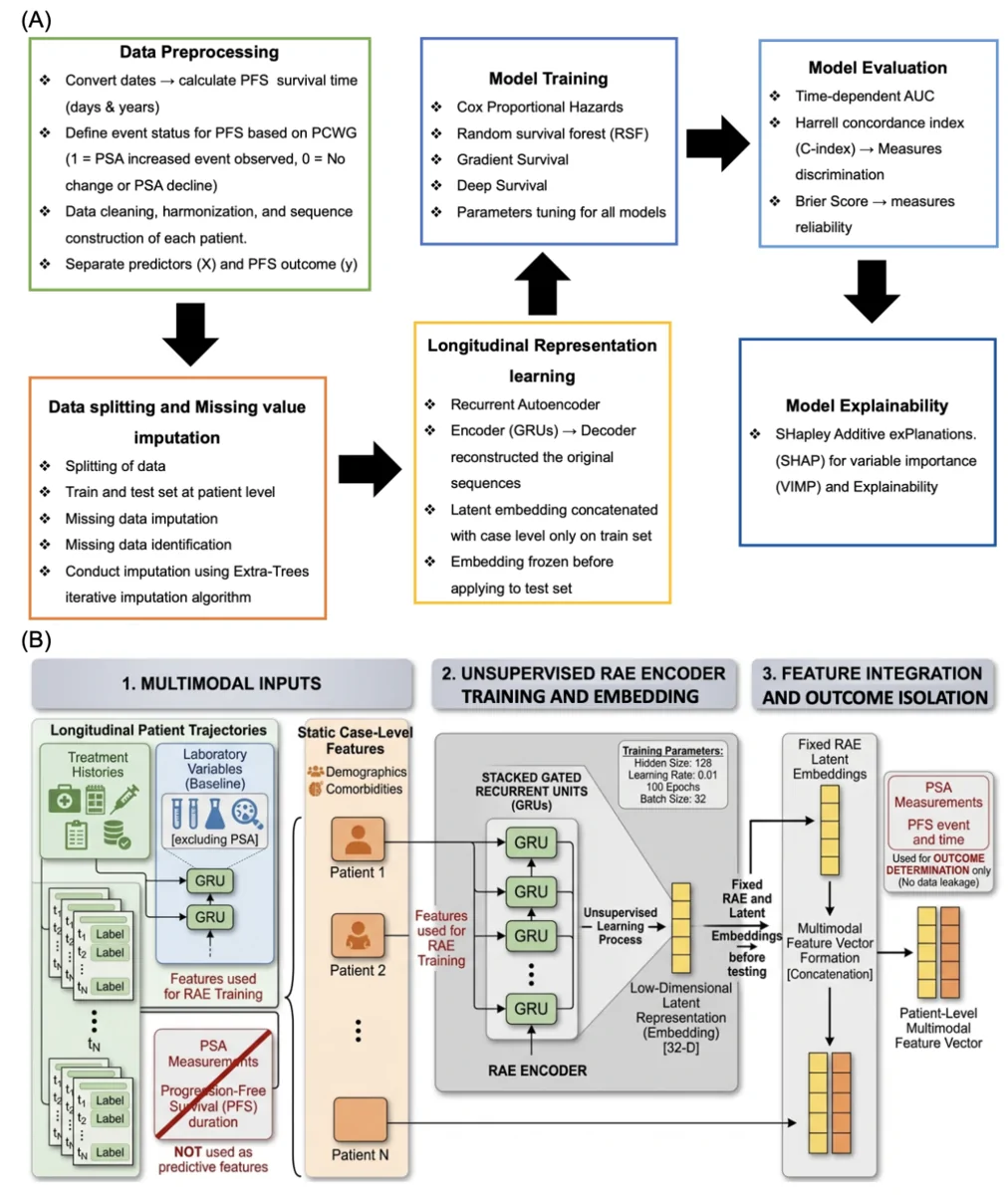
(A) Overview of the machine learning model development workflow for progression-free survival (PFS). (B) Recurrent autoencoder (RAE) architecture for longitudinal representation. AUC: area under the curve; GRU: gated recurrent unit; PCWG: Prostate Cancer Working Group; PSA: prostate-specific antigen.

### Longitudinal Representation Learning Using a Recurrent Autoencoder

A recurrent autoencoder (RAE) was trained on the training cohort to capture temporal patterns in laboratory and treatment histories (Figure 2B) [20]. PFS duration served as the primary temporal input, with all laboratory variables recorded as baseline measurements before each treatment decision. PSA measurements were used exclusively for PFS event determination according to biochemical progression criteria and were not incorporated as predictive features in model training. The PFS event and time were used only as indexing variables, not as features, to avoid learning in the autoencoder, thereby ensuring that the autoencoder learned purely unsupervised representations of longitudinal patient trajectories without exposure to the outcome. The RAE encoder, composed of stacked gated recurrent units (GRUs), transformed each patient’s sequence into a low-dimensional latent embedding, which the decoder then reconstructed to preserve the original temporal patterns. These latent embeddings were concatenated with case-level static features (eg, race and morbidities) to form a multimodal feature vector. To prevent data leakage, the RAE was trained exclusively on the training dataset, and the learned embeddings were fixed before application to the testing set. Hyperparameters included a hidden layer size of 128, an embedding dimension of 32, a learning rate of 0.01, 100 epochs, and a batch size of 32.

### Model Development

#### Traditional Cox Proportional Hazards Model

A Cox proportional hazards model was fitted to the training dataset as a conventional baseline for PFS analysis. The model was adjusted for clinically relevant covariates to quantify the association between predictors and the hazard of disease progression or death, defined as the instantaneous risk of a progression event at a given time, conditional on remaining progression-free up to that point [27,28]. Final covariate selection was guided by model fit, assessed using the log-likelihood ratio test. The proportional hazards assumption was examined using scaled Schoenfeld residuals and corresponding statistical tests to detect systematic departures from time-invariant covariate effects [19-32]. Predicted risk scores were generated for the testing dataset to facilitate direct comparison with ML-based PFS models.

#### ML Models

Three ML-based survival models were developed using an integrated feature set combining static clinical variables and latent temporal representations derived from the RAE. First, a random survival forest (RSF) model was trained as a nonparametric ensemble method to estimate cumulative hazard functions and rank predictors by importance [33]. Second, a gradient boosting survival (GBS) model (GBT-Survival) was implemented using a Cox partial likelihood loss function, enabling the modeling of nonlinear effects and complex interactions among covariates [34]. Third, a deep neural network–based Cox regression model (DeepSurv) was trained to capture nonlinear relationships between predictors and progression risk by optimizing the Cox partial likelihood [20]. Hyperparameter optimization for all models was performed using grid search cross-validation on the training dataset [35]. For the RSF, the explored parameter grid included the number of estimators (100, 200, 300), maximum depth (5, 7, 10), and minimum samples per split (10, 20, 30). For the GBS (GBT-Survival) model, the grid included learning rates (0.001, 0.01, 0.05, 0.1), the number of estimators (100, 200, 300), and maximum depth (3, 5, 7, 10). Optimal configurations, selected based on the highest concordance index, included RSF (maximum depth=7, minimum samples per split=10, 100 estimators), GBT-Survival (learning rate=0.001, maximum depth=10, 300 estimators), and DeepSurv (hidden layers=[32,35], dropout=0.1, rectified linear unit activation).

#### Model Evaluation and Explainability

Time-dependent area under the receiver operating characteristic curve (AUC) values could not be reliably estimated at 1, 2, or 3 years for any model because there were too few progression events and insufficient comparable patient pairs within these early periods to assess discrimination accurately. Model performance was assessed using time-dependent AUC values at the 4- and 5-year PFS horizons, together with Harrell concordance index (C-index) to evaluate discriminative ability [36]. Model robustness was examined using bootstrap resampling with 1000 iterations to derive CIs. Calibration was evaluated using the integrated Brier score (IBS) and calibration plots comparing predicted and observed progression-free survival probabilities. To enhance model interpretability, SHAP was applied to the best-performing RSF-Survival model, providing both global and patient-level insights into feature contributions to progression risk. SHAP summary, dependence, and force plots were used to visualize time-dependent predictor effects and identify key drivers of disease progression [19].

### Ethical Considerations

Ethics approval for this study was granted by the Medical and Health Research and Ethics Committee, Ministry of Health, Brunei Darussalam (MHREC/MOH/2024/22(1)), on January 25, 2025. All patient data were replaced with pseudonymous research IDs since we received the data from the hospital, and no personally identifiable information was accessed or retained within the analytic or modeling environment.

### Software Packages and Analytic Tools

All analyses were performed in Python using established open-source libraries, including *pandas* and *NumPy* for data manipulation; *scikit-survival* for Cox proportional hazards modeling, RSF, GBS analysis, concordance index estimation, and time-dependent AUC; *lifelines* and *pycox* for survival modeling (including DeepSurv); *PyTorch* (torch, torchvision, and torchaudio) for deep learning implementation; *fancyimpute* for iterative imputation; *scikit-learn* for data splitting and preprocessing (GroupShuffleSplit and StandardScaler); and *SHAP* for model interpretability.

## Results

### Characteristics and Univariable Analysis of Study Participants

The study included 212 patients at the case level, contributing a total of 478 longitudinal observations over 6 years (detailed participant characteristics are presented in Multimedia Appendix 2). PFS events were reported in 45 observations during follow-up. At the case level (n=212), PFS outcomes differed according to tumor-related factors, including prostate cancer stage and Gleason grade group, whereas other comorbid conditions did not differ significantly between PFS outcome groups. Furthermore, at the cohort level (478 observations), significant differences between observations with and without a PFS event were observed in calcium (*P*<.001), chloride (*P*<.001), sodium (*P*<.001), red blood cell count (*P*<.01), hemoglobin (*P*<.01), neutrophils (*P*<.01), eosinophils (*P*<.01), baseline PSA (*P*<.001), and posttreatment PSA (*P*<.001).

### Kaplan-Meier Curve for PFS

The PFS analysis with significant variables is presented using the Kaplan-Meier curves in Figure 3. The overall PFS curve demonstrates a steady decline over time, with the probability of remaining progression-free markedly decreasing after approximately 2 years of follow-up. Stratified analyses revealed significant differences across merged clinical stages (log-rank test, *P*=.0002), with advanced stages showing poorer PFS than earlier stages. Similarly, significant variation in PFS was observed among different prostate cancer treatment groups (log-rank test, *P*=.0009), indicating heterogeneity in outcomes depending on the therapeutic approach. Furthermore, PFS differed significantly according to place of treatment (log-rank test, *P*=.0033), with the inpatient treatment group demonstrating consistently lower survival probability over time.

**Figure 3:**
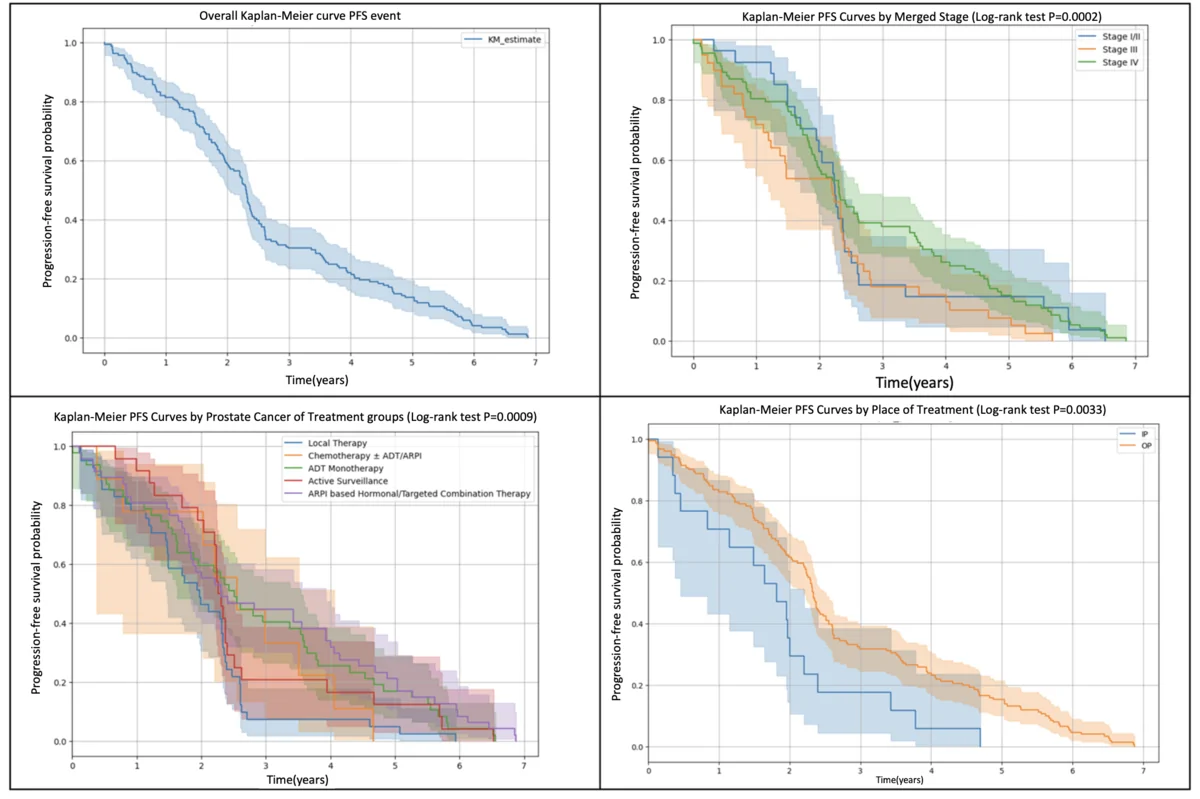
Kaplan-Meier curves for progression-free survival among prostate cancer patients at the Brunei Cancer Centre (TBCC) from January 2018 to December 2024. ADT: androgen deprivation therapy; ARPI: androgen receptor pathway inhibitor; IP: inpatients; OP: outpatients.

### Discriminative Performance of PFS Models

The discriminative performance, as measured by the time-dependent AUC at 1, 3, and 5 years (Figure 4), and the Harrell C-index (Table 1), was used to develop and compare 4 survival models. Evaluation of model performance at 4 and 5 years indicated that ML-based models outperformed the traditional Cox proportional hazards models. The Cox model demonstrated limited discriminative ability, with time-dependent AUCs of 0.706 and 0.833 at 4 and 5 years, respectively, along with a C-index of 0.531. In contrast, the tree-based models, RSF and GBT-Survival, showed markedly superior discrimination. The RSF model demonstrated strong discriminative performance at 4 and 5 years (AUCs of 0.941 and 0.917, respectively; C-index 0.906), while the deep survival model (AUCs of 0.941 at 4 years and 0.917 at 5-year; C-index 0.719) and the GBS model (AUCs of 0.765 at 4 years and 0.833 at 5-year, C-index 0.844) demonstrated moderate discriminative performance.

**Figure 4:**
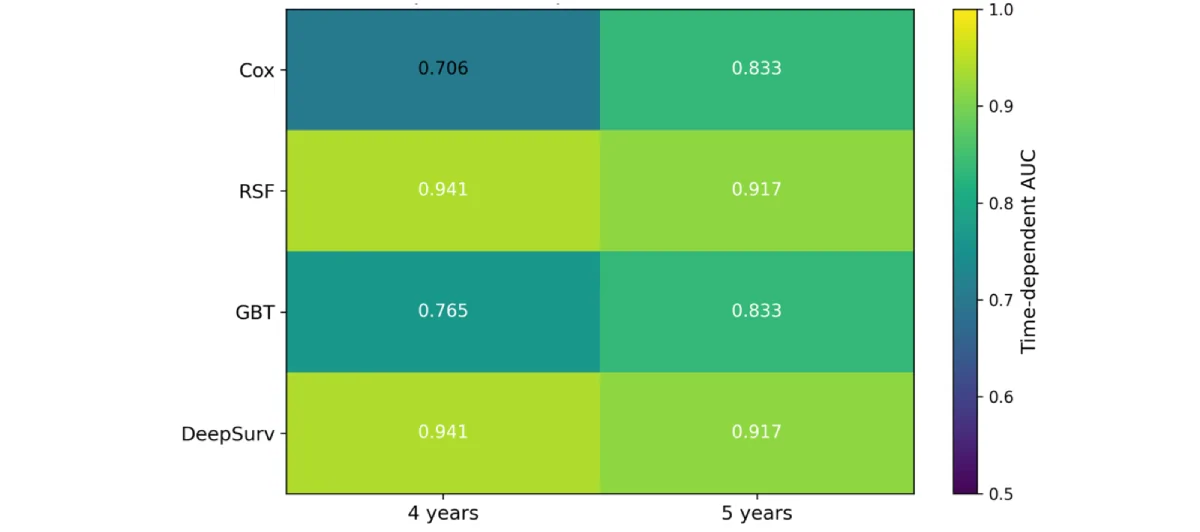
Heatmap of time-dependent area under the receiver operating characteristic curve (AUC) of progression-free survival (PFS) models, showing the relationship between predicted accuracy in fourth and fifth years. Cox: Cox proportional hazards model; DeepSurv: deep neural network–based Cox regression model; GBS: gradient boosting survival; RSF: random survival forest.

**Table 1:** Discriminative performance (time-dependent area under the receiver operating characteristic curve [AUC], Harrell concordance index [C-Index]) and calibration accuracy (integrated Brier score) of progression-free survival models.

Models	Time-dependent AUC		Harrell C-index	Integrated Brier score
	4 years	5 years		
Cox proportional hazards	0.706	0.833	0.531	0.0527
Random survival forest	0.941	0.917	0.906	0.0698
Gradient boosting survival	0.765	0.833	0.844	0.0590
Deep neural network survival	0.941	0.917	0.719	0.0887

### Calibration Performance Using IBS for PFS Model

Calibration performance of the PFS models was assessed using the IBS, where lower values indicate better agreement between predicted and observed outcomes (Table 1). Among the evaluated models, the Cox proportional hazards model demonstrated the best calibration accuracy with the lowest IBS of 0.0527. The GBS model demonstrated comparable calibration performance (IBS=0.0590), followed by the RSF model (IBS=0.0698). In contrast, the deep neural network survival model exhibited the highest IBS (IBS=0.0887), indicating comparatively poorer calibration despite its better discriminative performance.

Although similar time-dependent AUC values were observed between the RSF and deep neural network survival models at specific predefined time points, the limited number of events in the study may reduce the sensitivity of AUC to detect subtle performance differences between models. Therefore, we placed greater emphasis on the C-index and IBS, as these metrics provide a more comprehensive assessment of survival model performance across the entire follow-up period compared with isolated time-point AUC measurements. According to both discriminative and calibration performance, the RSF model demonstrated stable and well-calibrated risk prediction over time.

### Model Explainability Using SHAP for RSF Models for PFS

Model explainability for PFS prediction using the RSF was assessed using SHAP, providing both global and individual-level interpretability (Figure 5). At the global level, the SHAP feature importance heatmap (Figure 5A) indicated that biomedical and laboratory parameters, particularly baseline sodium, alanine aminotransferase (ALT), mean corpuscular hemoglobin (MCH) level, weight, and the treatment variable (Rx_C_D_Merged), were the most influential contributors to the model’s PFS predictions, while demographic variables showed comparatively lower impact. These features highlighted the dominant role of laboratory biomarkers in capturing progression risk in patients with prostate cancer. SHAP values reflect model attribution, not a causal effect.

At the individual level, with the sample patient, SHAP bar, force and waterfall plots (Figure 5B) illustrated how specific features contributed to the predicted PFS for a representative patient, demonstrating the direction and magnitude of each feature’s effect on expected survival time. ALT, sodium, MCH, platelet count, and treatment category increased the predicted risk of progression, whereas other features had minimal or neutral influence. These outputs offer clinical interpretability of how individual patient characteristics contribute to PFS predictions, thereby enhancing transparency, trust, and potential clinical utility in the prognosis of prostate cancer.

**Figure 5:**
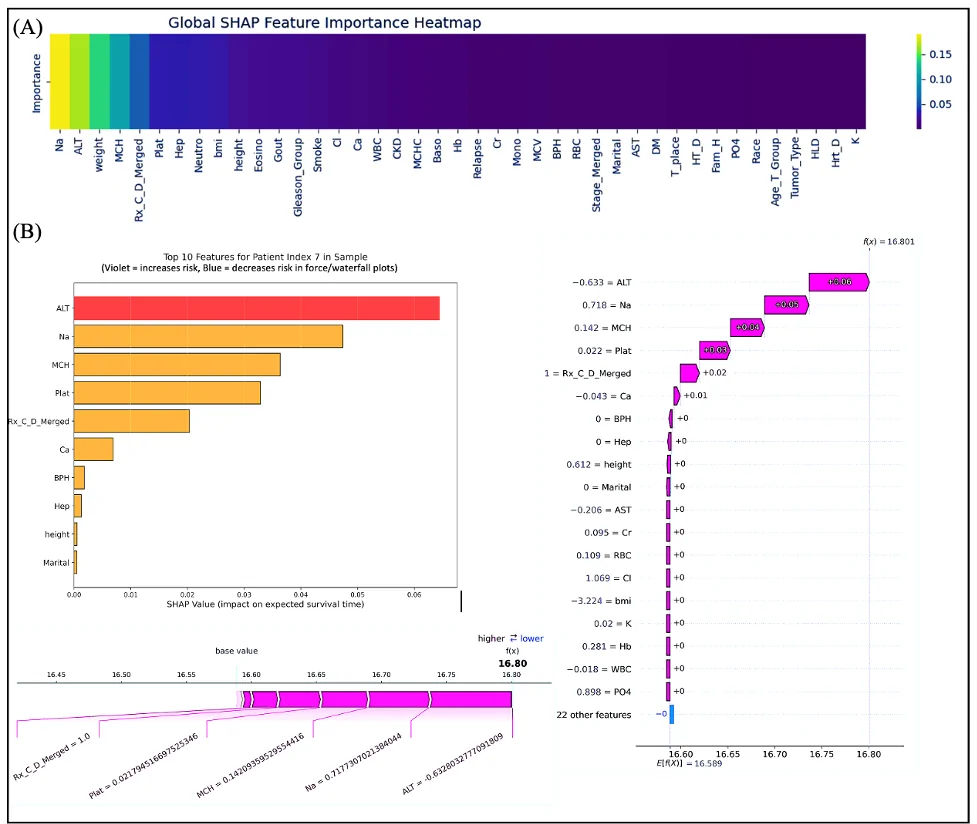
(A) Heatmap of global Shapley additive explanations (SHAP) feature importance for the random survival forest model. (B) Top 10 features by importance for an individual patient, bar, waterfall and force plots. ALT: alanine transaminase; BPH: benign prostatic hyperplasia; Ca: calcium; Cl: chloride; Hb: Hemoglobin; Hep: hepatitis; Na: sodium; Plat: platelet; PSA: prostate-specific antigen; Rx_C_D_Merged: treatment categories.

## Discussion

### Principal Findings

The study findings demonstrated that ML-based models showed improved internal discrimination compared with the traditional Cox proportional hazards model in predicting PFS among patients with prostate cancer, particularly at long-term horizons. While early PFS prediction at 1 to 3 years was not feasible due to insufficient progression events and limited comparable risk sets, robust discrimination emerged at 4 and 5 years. This reflects the delayed progression patterns and sluggish natural history of prostate cancer that are frequently seen in clinical practice [37-39]. Among the evaluated models, RSF showed the most balanced performance, achieving high time-dependent AUC values and stable calibration as measured by the IBS. These findings highlight the importance of aligning evaluation time points with disease biology and reinforce the limitations of early event prediction risk in prostate cancer cohorts [8,12,40].

Additionally, the study provided an explainability output from the RSF model using SHAP, which allows addressing the “black-box” barrier for clinical adoption of AI in oncology [19]. The SHAP-based global and individual explanations revealed that the laboratory biomarkers and treatment-related variables dominated the prediction of PFS. This aligns with emerging evidence that data-driven survival models capture complex, nonlinear associative predictors of risk that are often missed by conventional regression-based approaches [20,41]. Furthermore, the SHAP force and waterfall plots at the individual patient level enable clinicians to understand not only what the model predicts but how a particular prediction is made, which can further improve trust, auditability, and shared decision-making in clinical oncology [19,42,43].

Moreover, the application of a RAE to model the longitudinal cohort structure enabled effective compression of repeated observations into latent representations, providing a novel and efficient approach for managing complex temporal data while preserving clinically relevant information. The performance of RSF and GBS models in this cohort was consistent with earlier reports demonstrating the superiority of tree-based methods for cancer survival prediction [19,44]. Additionally, similar to previous studies on prostate cancer, RSF models outperformed Cox models when handling high-dimensional laboratory and longitudinal data, particularly in heterogeneous real-world cohorts [36,45]. The underperformance of Cox regression in this setting supports the rationale for exploring ML survival methods, particularly RSF, which can capture complex nonlinear interactions without proportional hazards assumptions. Although earlier studies reported strong short-term predictive performance, our findings suggest that PFS prediction in prostate cancer is inherently limited at early points [39,40]. The comparatively weaker calibration of the deep neural network survival model highlighted that higher discrimination does not necessarily translate into reliable absolute risk estimation.

According to the SHAP model, sodium, ALT, and hematological parameters such as MCH and platelet count emerged as influential predictors of PFS. Dysnatremia has been associated with cancer progression and systemic inflammation, while elevated ALT may reflect hepatic stress, metabolic dysfunction, or treatment-related toxicity, all of which have been linked to poorer oncological outcomes [46-48]. Moreover, studies have indicated that the aspartate aminotransferase to ALT ratio can be used as a prognostic biomarker for prostate tumor progression [49,50]. These findings support the growing recognition that routine laboratory biomarkers capture systemic physiological states relevant to cancer progression beyond tumor-centric variables alone [51]. However, the prominence of sodium and ALT in our SHAP analysis does not indicate that these markers are biologically superior to other prognostic factors; rather, within this dataset and modeling framework, they emerged as influential predictive contributors that improved the model’s ability to estimate risk.

This study has several limitations that should be acknowledged. First, the relatively small sample size, while sufficient for internal model development and evaluation, may restrict the generalizability of the results to other populations, particularly in light of regional differences in clinical practice and disease characteristics. This also limits the model’s ability to capture early PFS events. Performance estimates are likely optimistic due to limited event counts. Despite these limitations, our study offers important novel contributions that address key gaps in the literature. No prior study, to our knowledge, has collected the comprehensive range of variables included here or applied a sequential approach to define PFS, whereas most existing studies use a single PFS event per patient, limiting clinical applicability. Second, the retrospective nature of the study may introduce bias due to missing data, unmeasured confounding, and evolving treatment strategies over time. Furthermore, model performance was evaluated using a held-out testing dataset; although cross-validation was applied to reduce overfitting, the absence of external validation remains a limitation.

Third, although the analysis was retrospective and conducted at a single institution, the TBCC functions as the only comprehensive oncology facility in the country and is responsible for the management of nearly all prostate cancer cases nationwide. Nevertheless, the findings highlight the potential utility of ML models incorporating SHAP-based explainability for prognostic risk assessment in prostate cancer. Finally, radiographic progression according to PCWG criteria was not incorporated into the PFS analysis because of the limitations of the imaging data available for this cohort. Future research should include independent external validation and multi-institutional, multiomics datasets with larger and more diverse cohorts to strengthen the generalizability and robustness of these findings.

### Conclusions

Our study demonstrates that the ML-based survival models, particularly tree-based ensemble methods, substantially outperform the traditional Cox proportional hazards model in predicting PFS among patients with prostate cancer, especially at longer follow-up horizons. Among the evaluated approaches, the RFS model achieved the most balanced performance, combining strong long-term discrimination with stable calibration, thereby offering a robust framework for prognostic modeling in real-world prostate cancer cohorts. The integration of SHAP-based explainability addressed a key barrier to clinical adoption by providing transparent global and individual-level insights into model predictions highlighting the dominant role of laboratory biomarkers and treatment-related variables in driving PFS risk predictions in this cohort. The application of a RAE to encode longitudinal cohort data further enhanced the model’s ability to capture the complex temporal patterns while reducing dimensionality. These findings support the clinical potential of explainable ML models for personalized risk stratification in prostate cancer, while also emphasizing the need for external validation, larger multi-institutional, multiomics datasets, and prospective evaluation prior to routine clinical implementation.

## Appendix

Multimedia Appendix 1 Discriminative performance (time-dependent area under the curve, Harrell concordance index) and calibration accuracy (integrated Brier score) of progression-free survival models.

Multimedia Appendix 2 Characteristics of participants stratified by progression-free survival.

## Abbreviations

ALT: alanine aminotransferase
AUC: area under the receiver operating characteristic curve
C-index: concordance index
GBS: gradient boosting survival
GRU: gated recurrent unit
IBS: integrated Brier score
JPMC: Jerudong Park Medical Centre
MCH: mean corpuscular hemoglobin
ML: machine learning
PCWG: Prostate Cancer Working Group
PFS: progression-free survival
PSA: prostate-specific antigen
RAE: recurrent autoencoder
RSF: random survival forests
SHAP: Shapley additive explanations
TBCC: Brunei Cancer Centre

## Data Availability

The aggregate data and results supporting the findings of this study are included in the published article and its supplementary materials, where applicable. The underlying participant-level dataset is not available because of privacy and confidentiality considerations and to protect the anonymity of study participants. The complete analysis code is publicly available on GitHub [52].
