# Supplementary material for: Explainable Machine Learning–Based Prediction of Progression-Free Survival in Prostate Cancer: Retrospective Cohort Study
Source: JMIR Cancer. 2026 Oct 5;12:e91510. doi: 10.2196/91510 (PMC13680908; doi:10.2196/91510)
Supplement: Supplementary file 1 [file cancer_v12i1e91510_app1.docx]

| **Models** | **Time – Dependent AUC** | | **C-index (Harrell)** | **Integrated**  **Brier Score** |
| --- | --- | --- | --- | --- |
|  | 4 years | 5 years |  |  |
| Cox Proportional Hazard | 0.706 | 0.833 | 0.581 | 0.0527 |
| Random Survival Forest | 0.941 | 0.917 | 0.906 | 0.0698 |
| Gradient Boosting Survival | 0.765 | 0.833 | 0.844 | 0.0590 |
| Deep neural network Survival | 0.941 | 0.917 | 0.719 | 0.0887 |
