# Supplementary material for: Explainable Machine Learning–Based Prediction of Progression-Free Survival in Prostate Cancer: Retrospective Cohort Study
Source: JMIR Cancer. 2026 Oct 5;12:e91510. doi: 10.2196/91510 (PMC13680908; doi:10.2196/91510)
Supplement: Supplementary file 2 [file cancer_v12i1e91510_app2.docx]

| **Case level variables (n=212)** | **PFS (count % or mean (std))** | | **Univariate Test**  **(P-value)** |
| --- | --- | --- | --- |
|  | PFS without event | PFS event |  |
| **Age Group** | | | |
| 50-60 year | 17 (100.0%) | 0 (0.0%) | 0.1383 |
| 61-70 year | 66 (98.5%) | 1 (1.5%) |  |
| 71-80 year | 74 (93.7%) | 5 (6.3%) |  |
| >80 year | 44 (89.8%) | 5 (10.2%) |  |
| **Year of First Visit** | | | |
| 2024 | 31 (100.0%) | 0 (0.0%) | 0.0863 |
| 2023 | 38 (100.0%) | 0 (0.0%) |  |
| 2022 | 48 (82.8%) | 10 (17.2%) |  |
| 2021 | 17 (89.5%) | 2 (10.5%) |  |
| 2020 | 15 (68.2%) | 7 (31.8%) |  |
| 2019 | 17 (65.4%) | 9 (34.6%) |  |
| 2018 | 8 (44.4%) | 10 (55.6%) |  |
| **Race** | | | |
| Malay | 103 (79.2%) | 27 (20.8%) | 0.5576 |
| Chinese | 48 (82.8%) | 10 (17.2%) |  |
| Ethnic | 7 (77.8%) | 2 (22.2%) |  |
| Other | 7 (100.0%) | 0 (0.0%) |  |
| **Marital** | | | |
| Married | 139 (81.3%) | 32 (18.7%) | 1.0000 |
| Single/Others | 8 (80.0%) | 2 (20.0%) |  |
| **Family History** | | | |
| Absent | 129 (78.7%) | 35 (21.3%) | 0.2475 |
| Present | 42 (87.5%) | 6 (12.5%) |  |
| **Diabetes** | | | |
| Absent | 116 (83.5%) | 23 (16.5%) | 0.2158 |
| Present | 55 (75.3%) | 18 (24.7%) |  |
| **Hypertension** | | | |
| Present | 118 (83.1%) | 24 (16.9%) | 0.2734 |
| Absent | 53 (75.7%) | 17 (24.3%) |  |
| **Heart disease** | | | |
| Absent | 125 (78.6%) | 34 (21.4%) | 0.2694 |
| Present | 46 (86.8%) | 7 (13.2%) |  |
| **Hyperlipidaemia** | | | |
| Present | 93 (79.5%) | 24 (20.5%) | 0.7603 |
| Absent | 78 (82.1%) | 17 (17.9%) |  |
| **Gout** |  |  |  |
| Absent | 159 (82.4%) | 34 (17.6%) | 0.0854 |
| Present | 12 (63.2%) | 7 (36.8%) |  |
| **Hepatitis** | | | |
| Absent | 162 (80.6%) | 39 (19.4%) | 1.0000 |
| Present | 9 (81.8%) | 2 (18.2%) |  |
| **Benign Prostate Hyperplasia** | | | |
| Absent | 155 (81.2%) | 36 (18.8%) | 0.7985 |
| Present | 16 (76.2%) | 5 (23.8%) |  |
| **Chronic Kidney Disease** | | | |
| Absent | 155 (81.2%) | 36 (18.8%) | 0.7985 |
| Present | 16 (76.2%) | 5 (23.8%) |  |
| **Smoking history** | | | |
| Absent | 121 (79.1%) | 32 (20.9%) | 0.6951 |
| Present | 35 (83.3%) | 7 (16.7%) |  |
| Follow-up information |  |  | Median (IQR) |
| Median Follow up duration | 212 | 2.25 (1.30 – 3.52) | |
| **Gleason Group** | | | |
| Grade 5 | 60 (74.1%) | 21 (25.9%) | **< 0.01*** |
| Grade 4 | 29 (100.0%) | 0 (0.0%) |  |
| Grade 3 | 13 (92.9%) | 1 (7.1%) |  |
| Grade 2 | 25 (89.3%) | 3 (10.7%) |  |
| Grade 1 | 21 (95.5%) | 1 (4.5%) |  |
| **Cancer Staging** | | | |
| Stage IV | 95 (73.6%) | 34 (26.4%) | **< 0.001*** |
| Stage III | 39 (92.9%) | 3 (7.1%) |  |
| Stage I/II | 27 (100.0%) | 0 (0.0%) |  |
| **Tumour Type** | | | |
| Non specific | 115 (79.3%) | 30 (20.7%) | 0.4223 |
| mHSPC | 35 (89.7%) | 4 (10.3%) |  |
| mCRPC | 18 (75.0%) | 6 (25.0%) |  |
| nmCRPC | 3 (75.0%) | 1 (25.0%) |  |
| Weight in kg | 69.63 (15.78) | 62.91 (16.90) | **<0.01*** |
| Height cm | 142.99 (50.63) | 134.48 (57.21) | 0.0933 |
| **Cohort level variables (n=478)** | **PFS (count % or mean (std))** | | **Univariate Test (P-value)** |
|  | PFS without event | PFS event |  |
| **Age corresponding to Treatment** | | | |
| 46-60 year | 44 (93.6%) | 3 (6.4%) | 0.0620 |
| 61-70 year | 161 (93.6%) | 11 (6.4%) |  |
| 71-80 year | 157 (90.2%) | 17 (9.8%) |  |
| >80 year | 71 (83.5%) | 14 (16.5%) |  |
| **Patient visit** | | | |
| Outpatient | 350 (91.4%) | 33 (8.6%) | 0.3157 |
| Inpatient | 83 (87.4%) | 12 (12.6%) |  |
| **Relapse** | | | |
| No | 406 (90.2%) | 44 (9.8%) | 0.4487 |
| Yes | 27 (96.4%) | 1 (3.6%) |  |
| **Laboratory variables** | | | |
| Alanine transaminase (ALT) | 27.23 (23.60) | 22.21 (16.62) | 0.0954 |
| Aspartate transaminase (AST) | 27.55 (30.31) | 35.09 (63.72) | 0.7203 |
| Calcium (Ca) | 2.60 (5.64) | 2.14 (0.16) | **<0.001*** |
| Chlorine (Cl) | 105.27 (3.57) | 103.12 (3.47) | **<0.001*** |
| Creatinine (Cr) | 95.08 (67.76) | 83.94 (23.40) | 0.0642 |
| Urea (Ur) | 5.38 (1.91) | 4.55 (1.91) | 0.5276 |
| Potassium (K) | 4.63 (6.73) | 4.29 (0.53) | 0.9966 |
| Sodium (Na) | 139.77 (3.31) | 136.58 (4.18) | **<0.001*** |
| Phosphorus (PO4) | 1.03 (0.26) | 0.99 (0.29) | 0.2600 |
| Prostate Specific Antigen (PSA) baseline | 115.50 (296.60) | 292.62(531.83) | **<0.001*** |
| Prostate Specific Antigen (PSA) after treatment | 32.11 (120.72) | 161.34(384.55) | **<0.001*** |
| Red Blood Cell (RBC) | 4.22 (0.69) | 3.85 (0.88) | **<0.01*** |
| Haemoglobin (Hb) in g/dL | 122.55 (18.48) | 113.78 (19.89) | **<0.01*** |
| Mean corpuscular volume (MCV) | 88.58 (8.08) | 90.05 (7.52) | 0.4301 |
| Mean corpuscular haemoglobin (MCHC) | 330.52 (29.29) | 340.16 (79.58) | 0.5812 |
| White Blood Cell (WBC) | 7.88 (17.32) | 7.62 (2.39) | 0.2274 |
| Neutrophils (Neutro) | 4.37 (1.91) | 5.19 (2.02) | **<0.01*** |
| Monocytes (Mono) | 0.58 (0.30) | 0.63 (0.30) | 0.5455 |
| Eosinophils (Eosino) | 0.28 (0.30) | 0.16 (0.15) | **<0.01*** |
| Basophils (Baso) | 0.05 (0.06) | 0.04 (0.05) | 0.4398 |
| Platelets (Plat) | 243.01 (78.32) | 275.08(126.11) | 0.2252 |
